# Preserved miR-361-3p Expression Is an Independent Prognostic Indicator of Favorable Survival in Cervical Cancer

**DOI:** 10.1155/2018/8949606

**Published:** 2018-09-23

**Authors:** Shikai Liu, Lili Song, Hairong Yao, Liang Zhang, Dongkui Xu, Qian Li, Ying Li

**Affiliations:** Department of Obstetrics & Gynecology, Cangzhou Central Hospital, Hebei 061001, China

## Abstract

In this study, we aimed to assess the independent prognostic value of miR-361-3p in terms of overall survival (OS) and recurrence-free survival (RFS) in cervical cancer, as well as its possible regulative network. A retrospective analysis was performed by using data from the Cancer Genome Atlas-Cervical Cancer (TCGA-CESC). Results showed that decreased miR-361-3p expression was associated with lymphovascular invasion and poor responses to primary therapy. The patients with recurrence and the deceased cases had substantially lower miR-361-3p expression compared to their respective controls. By generating Kaplan-Meier curves of OS and RFS, we found that high miR-361-3p expression was associated with better survival outcome. More importantly, univariate and multivariate analysis confirmed that high miR-361-3p expression was an independent indicator of favorable OS (HR: 0.377, 95% CI: 0.233–0.608, *p* < 0.001) and RFS (HR: 0.398, 95% CI: 0.192–0.825, *p* = 0.013). By performing bioinformatic analysis, we identified 24 genes that were negatively correlated with miR-361-3p expression. Among the potential targeting genes, *SOST*, *MTA1*, *TFRC*, and *YAP1* are involved in some important signaling pathways modulating cervical cancer cell invasion, migration, and drug sensitivity. Therefore, it is meaningful to verify the potential regulative effect of miR-361-3p on the expression of these genes in the future.

## 1. Introduction

MicroRNAs (miRNAs) are a family of small, conserved, and noncoding RNAs that mainly exert regulative effect via repressing the transcription of targeting RNAs or inducing RNA degradation by binding to their 3′-UTR or 5′-UTR [1, 2]. *Homo sapiens* miR-361 stem-loop encodes two miRNAs, including miR-361-5p and miR-361-3p. A series of studies found that miR-361-5p acts as a tumor suppressor in multiple cancers. It suppresses lung cancer progression by targeting *FOXM1* [3]; inhibits hepatocellular carcinoma cell growth by targeting *CXCR6* [4]; decreases glycolytic metabolism, proliferation, and invasion of breast cancer by targeting *FGFR1* and *MMP1* [5]; and prevents the malignant progression of prostate cancer cells by targeting *STAT6* [6]. In comparison, the functional role of miR-361-3p in cancers was less studied. One recent study found that in nonsmall cell lung cancer (NSCLC), miR-361-3p could suppress tumor cell proliferation and metastasis by directly targeting *SH2B1* [7]. Decreased miR-361-3p expression in prostate secretion samples might be an important diagnostic marker of prostate cancer [8].

Dysregulated miRNAs have been implicated in the pathological development of cervical cancer. Our previous studies found that miR-21 could modulate the radiosensitivity of cervical cancer cells through targeting *LATS1* [9], while miR-375 could regulate the sensitivity by targeting *UBE3A* [10]. Some miRNAs have also been demonstrated as valuable prognostic markers in cervical cancer. For example, miR-155 upregulation was an independent prognostic indicator of overall survival for cervical cancer [11]. Downregulated serum level of miR-101 was an independent predictor of the unfavorable prognosis of cervical cancer [12].

However, the prognostic value of miR-361-3p in cervical cancer has not been explored. In this study, we assessed the independent prognostic value of miR-361-3p in terms of overall survival (OS) and recurrence-free survival (RFS) in cervical cancer, by using data from the Cancer Genome Atlas-Cervical Cancer (TCGA-CESC). In addition, we also examined the possible regulative network of miR-361-3p by performing a bioinformatic analysis.

## 2. Materials and Methods

### 2.1. Human Tissue Specimens

Cervical cancer tissues were obtained from 31 patients with primary cervical squamous cell carcinoma (SCC) who underwent radical trachelectomy or hysterectomy with pelvic lymph node dissection from July 2014 through December 2016 in Cangzhou Central Hospital. The mean age was 46.3 years (range, 31 to 68 years). Among the 31 cases, 23 cases were International Federation of Gynecology and Obstetrics (FIGO) stage I and 8 were stage II. All of the patients never received preoperative radiotherapy and/or chemotherapy before the tissues were collected. Normal cervical tissues with high-risk human papilloma virus (HR-HPV) infection (*N* = 19) or without HPV infection (*N* = 25) were obtained from patients who had trachelectomy or hysterectomy due to nonmalignant conditions. The tissue samples were immediately snap-frozen in liquid nitrogen and stored at −80°C until RNA extraction. This study was approved by the Ethics Committee of Cangzhou Central Hospital.

### 2.2. QRT-PCR Analysis of miR-361-3p Expression

Total RNAs in tissue samples were extracted using the TRIzol reagent (Invitrogen, Carlsbad, CA, USA) according to manufacturer's instructions. miRNA specific cDNA was synthesized using the stem-loop primers (5′-GTC GTA TCC AGT GCA GGG TCC GAG GTA TTC GCA CTG GAT ACG AAA TCA GAA T-3′) and the TaqMan microRNA reverse transcription kit (Applied Biosystems, Foster City, CA, USA). miR-361-3p expression was quantified by qRT-PCR, using TaqMan microRNA assays (Applied Biosystems). All PCR reactions were performed using an ABI Prism 7900 system (Applied Biosystems). The results were calculated using the 2^-ΔΔCT^ methods.

### 2.3. Retrieving of Data in TCGA-CESC

The level-3 data in TCGA-CESC was obtained by using the UCSC Xena Browser (https://xenabrowser.net/). In this cohort, 308 patients with primary cervical cancer were included. Among these patients, 306 cases had miR-361-3p expression measured by RNAseq (IlluminaHiseq), 293 cases had intact OS data recorded, and 254 cases belong to squamous cell carcinoma (SCC). The clinicopathological data of the patients with intact OS data were downloaded for survival-related analysis, including age at initial pathologic diagnosis, histology, tumor grade, clinical stage, lymphovascular invasion indicator, primary therapy outcomes, the history of radiation therapy, targeted molecular therapy and postoperative drugs, recurrence status, and living status.

### 2.4. Bioinformatic Analysis of the Possible Targets of miR-361-3p in Cervical Cancer

The possible targets of miR-361-3p were predicted using TargetScan 7.1, by setting the cumulative weighted context++ score ≤ −0.3 as the threshold. The genes that were negatively correlated with miR-361-3p in TCGA-CESC were identified by setting Pearson's *r* value ≤ −0.2 as the threshold. The overlapping subset between the negatively correlated genes and the predicted target genes was identified. Then, the genes were loaded into ClueGO to analyze their functional annotations in Gene Ontology (GO). Only pathways with *p* value < 0.05 were included.

### 2.5. Statistical Analysis

Statistical analysis was performed by using GraphPad Prism 6.0 (GraphPad Inc., La Jolla, CA, USA) or SPSS 19.0 software package (SPSS Inc., Chicago, IL, USA). Welch's unequal variances *t*-test was conducted to compare the difference of miR-361-3p expression in different groups. Receiver operating characteristic (ROC) analysis for death and recurrence detection was performed to identify the best cutoff (Youden index) for miR-361-3p expression in Kaplan-Meier curves. Log-rank test was applied to evaluate the significance of the difference between the survival curves. Fisher's exact test was used to assess the association between miR-361-3p expression and the clinicopathological parameters. Univariate and multivariate Cox regression analysis was conducted to determine the independent prognostic value of miR-361-3p expression in terms of OS and RFS. *p* < 0.05 was considered statistically significant.

## 3. Results

### 3.1. Patients with Different Survival Outcomes Have Varying miR-361-3p Expressions

**Figure 1:**
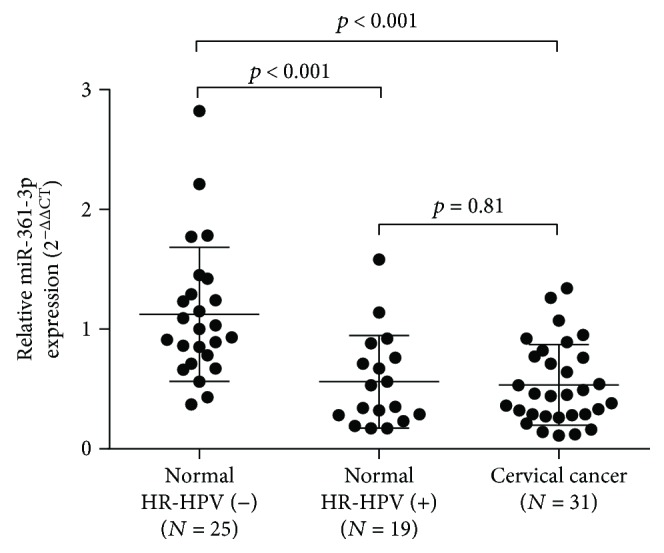
miR-361-3p expression profile. miR-361-3p expression was significantly decreased in HR-HPV(+) normal cervical tissues (*N* = 19) and in cervical cancer tissues (*N* = 31) compared to that in HR-HPV(−) normal cervical tissues (*N* = 25).

Using normal and tumor tissue samples, we found that the normal tissues with high-risk human papillomavirus (HR-HPV) infection (*N* = 19) had significantly decreased miR-361-3p expression compared with the normal tissues without the infection (*N* = 25) (*p* < 0.001) (Figure 1). However, we failed to identify significant difference in miR-361-3p expression between HR-HPV (+) normal tissues and cancerous cervical cancer tissues (Figure 1). Using data from TCGA, we also compared miR-361-3p expression in 31 types of cancer with miRNA-seq data. Results showed that in colorectal cancer, thyroid cancer, and non-small-cell lung cancer, miR-361-3p expression tends to be downregulated in cancer tissues than in adjacent normal tissues (Supplementary Figure 1, red frames), while the opposite trend was observed in kidney clear cell carcinoma, stomach adenocarcinoma, bladder urothelial carcinoma, uterine corpus endometrioid carcinoma, and prostate adenocarcinoma (Supplementary Figure 1, green frames). These findings suggest the miR-361-3p expression change might be cancer specific.

**Figure 2:**
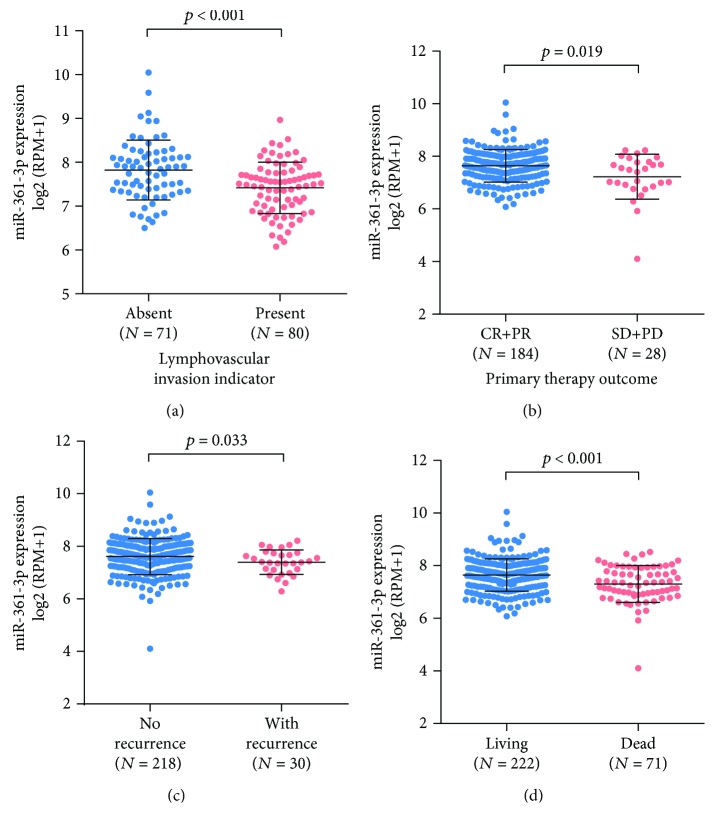
Decreased miR-361-3p was associated with malignant behaviors of cervical cancer. (a, b) Comparison of miR-361-3p expression in cervical cancer patients with or without lymphovascular invasion (a) or in patients with response to primary therapy (CR and PR) or with no response to primary therapy (SD and PD) (b). (c, d) Comparison of miR-361-3p expression in cervical cancer patients with or without recurrence (c) or in deceased or living patients (d). CR: complete remission; PR: partial remission; SD: stable disease, and PD: progressive disease.

Then, we explored miR-361-3p expression between patients with different clinicopathological features. Results showed that the cases with lymphovascular invasion (*N* = 80) had significantly decreased miR-361-3p expression compared to the lymphovascular negative counterparts (*N* = 71, *p* < 0.001) (Figure 2(a)). The patients with stable disease (SD) or progressive disease (PD) (*N* = 28) also had significantly lower miR-361-3p expression compared to the cases with complete remission (CR) or partial remission (PR) (*N* = 184) (*p* = 0.019, Figure 2(b)). However, no significant difference was observed between stage I/II and stage III/IV diseases (Supplementary Figure 2). Then, we examined the discrepancy in miR-361-3p expression between the patients with different survival outcomes. Results showed that the cases with recurrence and the deceased cases had substantially lower miR-361-3p expression compared to their respective controls (*p* = 0.033 and *p* < 0.001, Figures 2(c) and 2(d)).

### 3.2. Preserved miR-361-3p Expression Was an Independent Indicator of Favorable OS and RFS in Cervical Cancer

**Figure 3:**
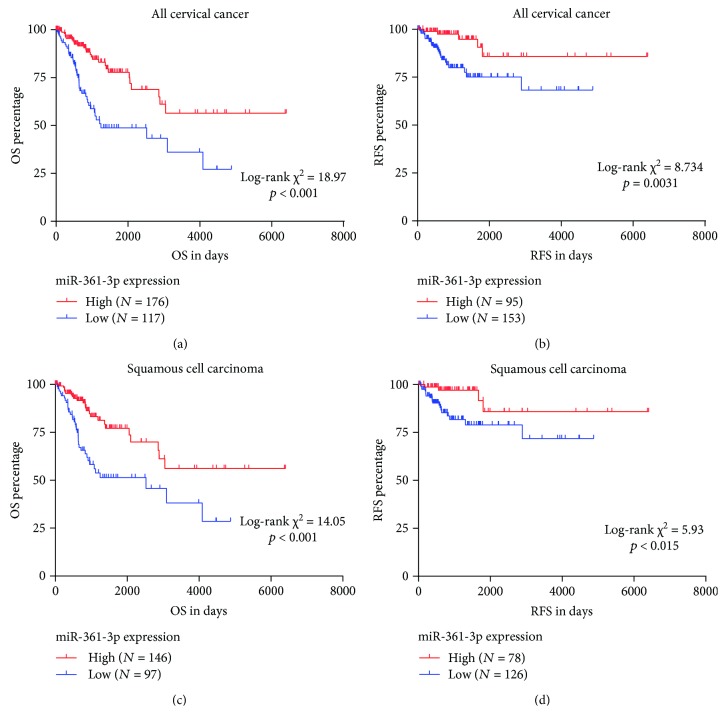
Kaplan-Meier curves of OS and RFS in patients with cervical cancer. (a–d) Kaplan-Meier curves of OS (a, c) and RFS (b and d) in all cervical cancer patients (a, b) and in patients with cervical squamous cell carcinoma (c, d). Patients were grouped according to the optimal cutoff of miR-361-3p expression in the ROC curves for death and recurrence detection.

Since the patients with different survival outcomes had different miR-361-3p expression, we decided to explore whether this miRNA is a prognostic marker in cervical cancer. By generating Kaplan-Meier curves of OS and RFS, we found that the low miR-361-3p expression group had significantly worse OS (*p* < 0.001) and RFS (*p* = 0.0031) compared to the high miR-361-3p expression group (Figures 3(a) and 3(b)). Since squamous cell carcinoma is the major subtype of cervical cancer, we also examined whether the associations were significant in this histological subtype. Using the same cutoff identified in Figures 3(a) and 3(b), we confirmed that low miR-361-3p expression was also significantly associated with unfavorable OS (*p* < 0.001) and RFS (*p* = 0.015) in squamous cell carcinoma (Figures 3(c) and 3(d)).

Then, we tried to investigate the independent prognostic value of miR-361-3p in cervical cancer. The associations between miR-361-3p expression and the clinicopathological parameters were summarized in Table 1. Results showed that the low miR-361-3p expression group had a significantly higher ratio of recurrence (17/96 vs. 13/152, *p* = 0.044) and death (43/117 vs. 28/176, *p* < 0.001) compared to the high miR-361-3p expression group (Table 1). No significant association was observed in other parameters, suggesting that these two groups were comparable. By performing univariate and multivariate analysis by using COX regression model, we found that early clinical stages (I/II), negative lymphovascular invasion, and high miR-361-3p expression (HR: 0.377, 95% CI: 0.233–0.608, *p* < 0.001) were independent indicators of favorable OS in cervical cancer (Table 2). By using RFS as the survival indicator, we also found that high miR-361-3p expression was an independent indicator of favorable RFS (HR: 0.398, 95% CI: 0.192–0.825, *p* = 0.013) in cervical cancer, after adjustment of lymphovascular invasion indicator (Table 3).

### 3.3. Bioinformatic Analysis of the Possible Regulative Network of miR-361-3p in Cervical Cancer

**Figure 4:**
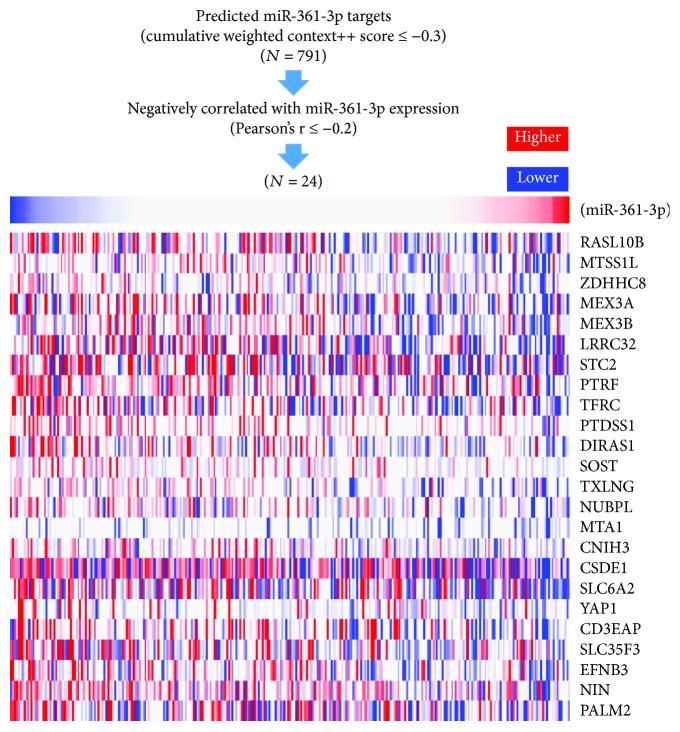
Screening of the genes negatively correlated with miR-361-3p in cervical cancer. Screening process showing the potential miR-361-3p targeting genes with negative correlation with miR-361-3p expression in cervical cancer tissues (*N* = 306).

**Figure 5:**
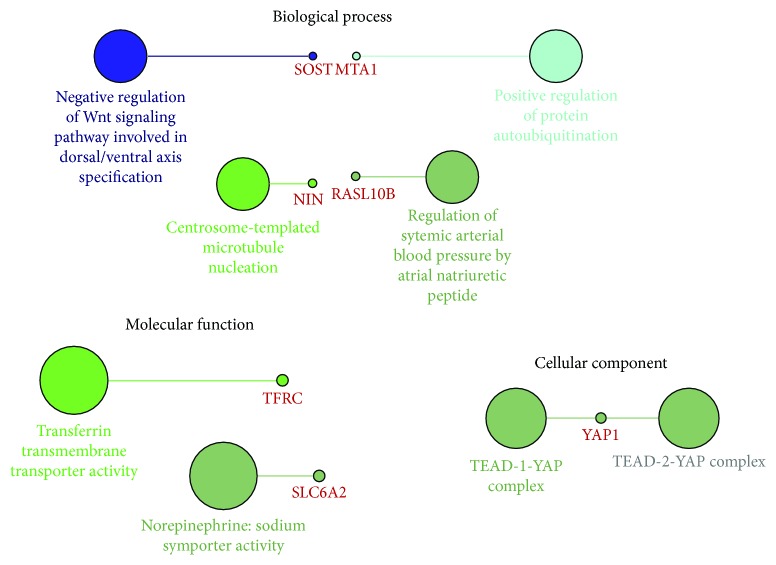
Functional annotations of the possible targets of miR-361-3p in cervical cancer in Gene Ontology.

Since one miRNA can participate in the regulation of multiple signaling pathways via targeting different mRNAs, we then explored the potential regulative network of miR-361-3p in cervical cancer. Using online prediction software, we identified 791 predicted target genes of miR-361-3p, by setting cumulative weighted context++ score ≤ −0.3 as the threshold (Figure 4). Then, we examined their coexpression with miR-361-3p in cervical cancer via their Pearson's *r* value. By setting Pearson's *r* value ≤ −0.2 as the threshold, we identified 24 genes that were negatively correlated with miR-361-3p expression (Figure 4). To explore the potential signaling pathways in which miR-361-3p might be involved in, the 24 genes were subjected to the analysis of functional annotations in Gene Ontology (GO). Results showed that in biological process, *SOST* was involved in the negative regulation of Wnt signaling pathway, *MTA1* was involved in the positive regulation of protein autoubiquitination, *NIN* was involved in centrosome-templated microtubule nucleation, and *RASL10B* was involved in the regulation of systemic arterial blood pressure by atrial natriuretic peptide (Figure 5). In molecular function, *TFRC* was involved in transferrin transmembrane transporter activity, while *SLC6A2* was involved in norepinephrine:sodium symporter activity (Figure 5). In cellular component, *YAP1* was involved in TEAD-1-YAP complex (Figure 5).

## 4. Discussion

There have been very limited studies that reported the regulative effect of miR-361-3p in cancers. One previous study found that miR-361-3p might act as a tumor suppressor in NSCLC and its antioncogenic activity may be due to its inhibition of the target gene *SH2B1* [7]. miR-361-3p downregulation may promote lymph node metastasis of T1-stage colorectal cancer via the upregulation of *E2F1* or *RAP2B* expression [13]. In this study, we observed significantly lower miR-361-3p expression in HR-HPV-positive cases than in the negative cases. By analyzing the association between miR-361-3p expression and the clinicopathological parameters of cervical cancer, we found that preserved miR-361-3p expression was associated with better primary therapy responses and a lower ratio of lymphovascular invasion, recurrence, and death. However, no significant difference was observed between stage I/II and stage III/IV diseases, in terms of miR-361-3p expression. Therefore, we hypothesized that miR-361-3p downregulation might be related to HR-HPV infection. Its downregulation might consistently involve malignant transformation, progression, and therapeutic responses of cervical cancer. However, future molecular studies are required to verify these hypotheses. By generating Kaplan-Meier curves of OS and RFS, we found that high miR-361-3p expression was associated with better survival outcome. More importantly, univariate and multivariate analysis confirmed that high miR-361-3p expression was an independent indicator of favorable OS (HR: 0.377, 95% CI: 0.233–0.608, *p* < 0.001) and RFS (HR: 0.398, 95% CI: 0.192–0.825, *p* = 0.013). These findings suggest that miR-361-3p might be a valuable prognostic biomarker of cervical cancer.

One miRNA might be involved in the regulation of multiple signaling pathways via targeting different mRNAs, and the targeting mRNAs might be cancer specific. To guide further exploration of the possible targets of miR-361-3p in cervical cancer, we performed bioinformatic analysis to identify the highly possible targets. Our results found 24 predicted genes that were negatively correlated with miR-361-3p expression in cervical cancer. Among the genes, *SOST* is involved in the negative regulation of the Wnt signaling pathway, which is a critical signaling pathway modulating cervical cancer cell proliferation, migration, and angiogenesis [14, 15]. *MTA1* is involved in the positive regulation of protein autoubiquitination. In fact, this gene has been characterized as an important oncogene facilitating cervical cancer progression and metastasis [16, 17]. Knockdown of *MTA1* partly restores p53 and E-cadherin expression in cervical cancer cells and subsequently decreases their invasion, migration, and adhesion capabilities [18]. *TFRC* is significantly upregulated in cervical cancer and is considered as a candidate gene associated with the invasion of cervical cancer [19]. As an important component of the TEAD-1-YAP complex, *YAP1* upregulation in cytoplasm is associated with histologic grading, lymph node metastasis, and recurrence of cervical SCC [20], and its nuclear overexpression is associated with shorter OS and disease-free survival of cervical adenocarcinoma [20]. *YAP1* inhibition is associated with upregulation of p21 and p27 cell-cycle inhibitors and results in decreased cell proliferation, cell migration, anchorage-independent growth, and *in vivo* tumorigenic potential [21]. Besides, *YAP1* silencing also increases the sensitivity of cervical cancer cells to cisplatin treatment [21]. Mechanistically, YAP expression is correlated with human papillomavirus (HPV) integration status and is required for the upregulation of TGF-alpha, amphiregulin (AREG), and EGFR, thereby forming a positive signaling loop to drive cervical cancer cell proliferation [22, 23]. In this study, we also found that miR-361-3p might be involved in the regulation of cervical cancer invasion and primary therapy responses. Therefore, it is meaningful to verify the potential regulative effect of miR-361-3p on the expression of these genes in the future.

## 5. Conclusion

Preserved miR-361-3p expression is an independent prognostic indicator of favorable OS and RFS in cervical cancer. It is meaningful to verify the potential regulative effect of miR-361-3p on the expression of *SOST*, *MTA1*, *TFRC*, and *YAP1* in the future.

## Figures and Tables

**Table 1:** Comparison of the parameters between high and low miR-361-3p expression groups.

Parameters	miR-361-3p expression		*p* value
	High (*N* = 176)	Low (*N* = 117)	
Age (mean ± SD)	47.91 ± 13.32	48.51 ± 14.48	0.72
Histology			
Squamous cell carcinoma	142	101	0.082
Adenocarcinoma	32	12	
Adenosquamous carcinoma	2	4	
Grade			
G1/G2	90	57	0.71
G3/G4	70	49	
No data	16	11	
Clinical stage			
I/II	135	90	0.88
III/IV	38	24	
No data	3	3	
Lymphovascular invasion indicator			
Absent	49	21	0.093
Present	45	35	
No data	82	61	
Radiation therapy			
No	45	32	0.89
Yes	108	73	
Null	23	12	
Targeted molecular therapy			
No	38	29	0.36
Yes	84	48	
No data	54	40	
Postoperative drugs			
No	56	34	0.89
Yes	81	46	
No data	39	37	
Recurrence status			
No	139	79	0.044
Yes	13	17	
Null	24	21	
Living status			
Living	148	74	<0.001
Dead	28	43	

G1: well differentiated (low grade); G2: moderately differentiated (intermediate grade); G3: poorly differentiated (high grade); G4: undifferentiated (high grade).

**Table 2:** Univariate and multivariate analysis of OS in patients with cervical cancer.

Parameters	Univariate analysis				Multivariate analysis			
	*p*	HR	95% CI (lower/upper)		*p*	HR	95% CI (lower/upper)	
*Age*	0.061	1.017	0.999	1.035				
*Histology*								
Squamous cell carcinoma		1.000						
Adenocarcinoma	0.869	0.945	0.483	1.850				
Adenosquamous carcinoma	0.642	1.604	0.219	11.744				
*Clinical stage*								
III/IV vs. I/II	0.001	2.248	1.373	3.679	0.066	1.610	0.970	2.674
*Lymphovascular invasion indicator*								
Absent vs. present	0.002	0.098	0.023	0.415	0.002	0.105	0.025	0.449
*Radiation therapy*								
No vs. yes	0.237	0.700	0.388	1.264				
*Targeted molecular therapy*								
No vs. yes	0.701	1.114	0.643	1.930				
*Postoperative drugs*								
No vs. yes	0.384	0.765	0.419	1.397				
*miR-361-3p expression*								
High vs. low	<0.001	0.362	0.225	0.583	<0.001	0.377	0.233	0.608

**Table 3:** Univariate and multivariate analysis of RFS in patients with cervical cancer.

Parameters	Univariate analysis				Multivariate analysis			
	*p*	HR	95% CI (lower/upper)		*p*	HR	95% CI (lower/upper)	
*Age*	0.733	1.005	0.978	1.033				
*Histology*								
Squamous cell carcinoma		1.000						
Adenocarcinoma	0.426	1.441	0.586	3.545				
Adenosquamous carcinoma	0.222	3.535	0.466	26.821				
*Clinical stage*								
III/IV vs. I/II	0.974	1.015	0.415	2.486				
*Lymphovascular invasion indicator*								
Absent vs. present	0.006	0.128	0.029	0.562	0.007	0.131	0.030	0.572
*Radiation therapy*								
No vs. yes	0.399	0.679	0.276	1.670				
*Targeted molecular therapy*								
No vs. yes	0.820	1.098	0.489	2.467				
*Postoperative drugs*								
No vs. yes	0.499	0.694	0.241	2.000				
*miR-361-3p expression*								
High vs. low	0.025	0.578	0.358	0.932	0.013	0.398	0.192	0.825

## Data Availability

All relevant data were included in this article. The level-3 data in TCGA-OV used in this study can be accessed via the UCSC Xena Browser (https://xenabrowser.net/).

## Supplementary Materials

Supplementary Materials Supplementary Figure 1: the expression profile of miR-361-3p in tumors and adjacent normal tissues in TCGA. Supplementary Figure 2: miR-361-3p expression between stage I/II and stage III/IV cervical cancer.
