# Supplementary figures and images for: Preserved miR-361-3p Expression Is an Independent Prognostic Indicator of Favorable Survival in Cervical Cancer
Source: Dis Markers. 2018 Sep 23;2018:8949606. doi: 10.1155/2018/8949606 (PMC6174793; doi:10.1155/2018/8949606)

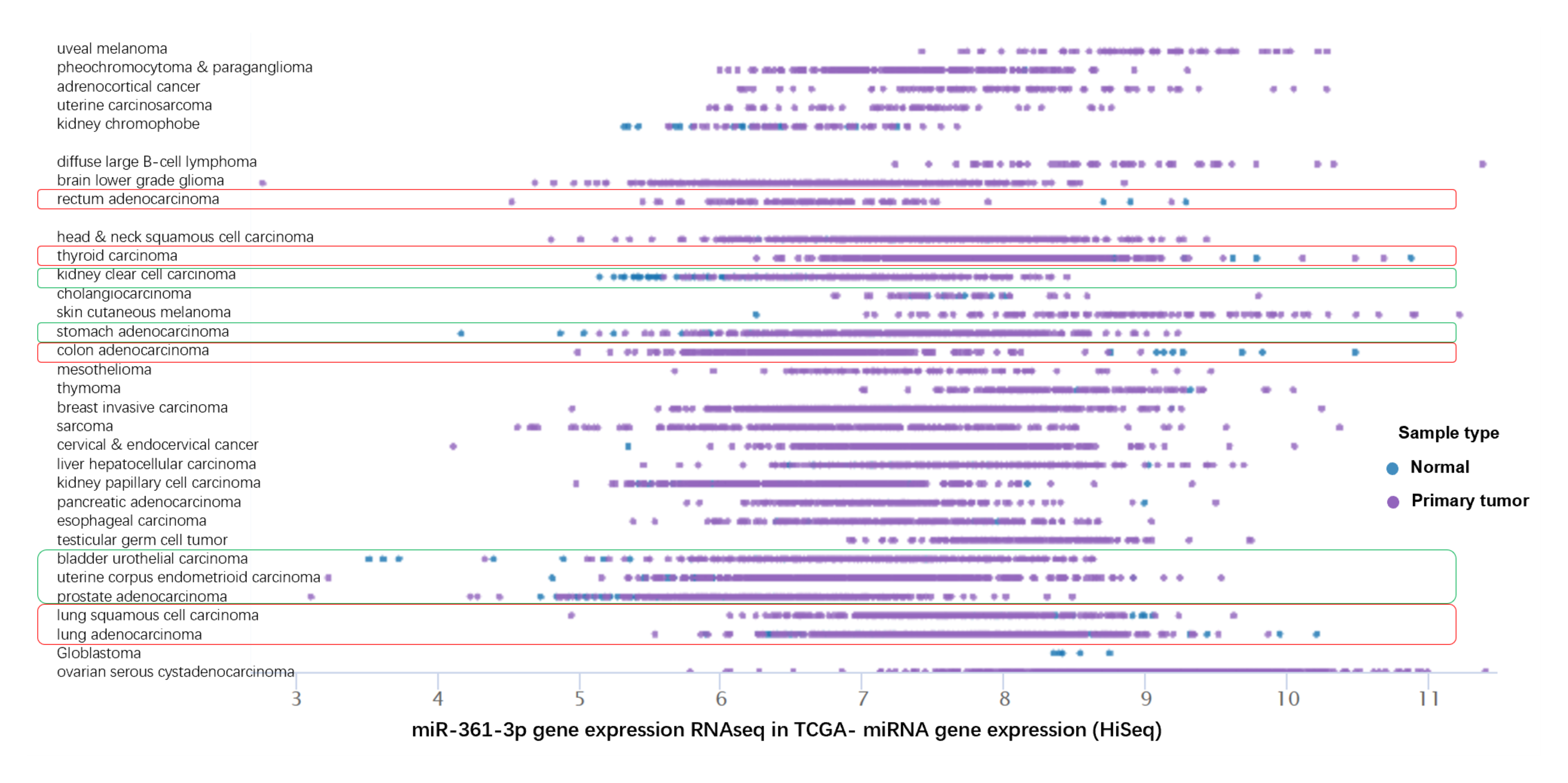

Supplement: Supplementary Materials — Supplementary Figure 1: the expression profile of miR-361-3p in tumors and adjacent normal tissues in TCGA. Supplementary Figure 2: miR-361-3p expression between stage I/II and stage III/IV cervical cancer. [file 8949606.f1.zip › Supplementary figure 1.tif]

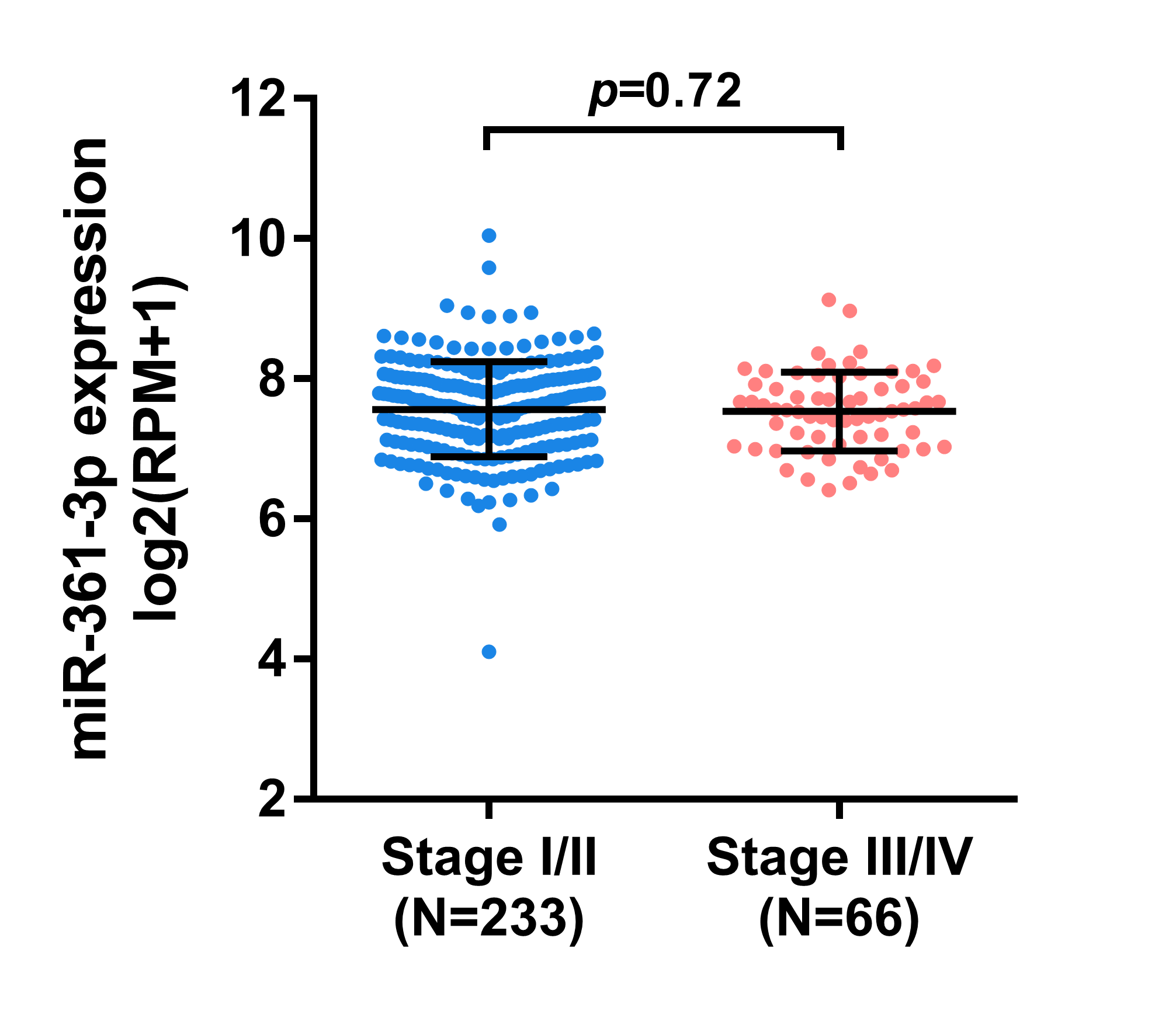

Supplement: Supplementary Materials — Supplementary Figure 1: the expression profile of miR-361-3p in tumors and adjacent normal tissues in TCGA. Supplementary Figure 2: miR-361-3p expression between stage I/II and stage III/IV cervical cancer. [file 8949606.f1.zip › Supplementary figure 2.tif]
